# How do worry and clinical status impact working memory performance? An experimental investigation

**DOI:** 10.1186/s12888-020-02694-x

**Published:** 2020-06-19

**Authors:** Judith Held, Andreea Vîslă, Richard E. Zinbarg, Christine Wolfer, Christoph Flückiger

**Affiliations:** 1 Department of Psychology, Psychological Interventions and Psychotherapy, University of Zurich, Zurich, Switzerland; 2 Department of Psychology, Northwestern University, Evanston, IL USA

**Keywords:** Worry, Working memory, Anxiety disorders, Generalized anxiety disorder, Cognitive performance

## Abstract

**Background:**

Previous research has suggested that worry is negatively associated with working memory performance. However, it is unclear whether these findings would replicate across different worry levels and in individuals with anxiety and depressive disorders (i.e. clinical statuses).

**Method:**

One-hundred-thirty-eight participants performed a two-block working memory task (150 trials per block). Based on participants` current clinical status, four groups were considered (generalised anxiety disorder group: *n* = 36; clinical group with another anxiety or mood disorders: *n* = 33; subclinical group: *n* = 27; control group: *n* = 42). Trait worry levels were collected from all of the participants. Working memory performance was measured as accuracy and reaction time.

**Results:**

During the first block, higher worry scores were significantly associated with longer reaction times. Moreover, the generalised anxiety disorder group, clinical group, and subclinical groups demonstrated significantly longer reaction times compared to the control group in Block 1, when age was controlled for. From Block 1 to Block 2, all of the participants demonstrated a significant decrease in accuracy and reaction time, regardless of worry level or clinical status.

**Conclusion:**

The results indicate that higher worry levels negatively impact WM processing efficiency. Moreover, when age was controlled for, we found participants` clinical status to be linked with WM impairments. The results highlight the relevance of investigating the impact of different worry levels on cognitive processes across clinical and non-clinical populations.

## Background

Worry is commonly experienced by a vast majority of people, including healthy children and adults of all age groups, as well as those suffering from anxiety and mood disorders [1]. In its extreme form, pathological worry has been described as a chain of thoughts that is laden with negative affect and perceived as relatively uncontrollable [2]. Although pathological worry is one of the cardinal features of generalized anxiety disorder (GAD [3];), it has also been found to be involved in other anxiety and mood disorders [1, 4].

Moreover, the high comorbidity rates among anxiety and depressive disorders point to shared underlying cognitive, emotional and behavioural processes across specific disorders [5]. In line with this, the Research Domain Criteria era (RDoC [6, 7];) proposed worry to be a transdiagnostic process that cuts across the traditional diagnostic categories. Moreover, the RDoC suggests that mental disorders are considered as problems in psychological as well as neurological systems and within that framework, disrupted cognitive processes are thought to play an important role in the maintenance of pathological worry in anxiety and depressive disorders [8].

A growing amount of studies suggests that worry binds cognitive resources, particularly in working memory (WM, [9]). The WM is a prominent system central to cognitive functioning [10]. It has a limited capacity and is, therefore, restricted in how much information can be actively held in WM [11]. A prominent theory attempting to explain how worry affects WM performance is the Attentional Control Theory (ACT [12];). It proposes that worry acts as a secondary task and thereby occupies available WM resources otherwise spent on the ongoing, primary task. That is, worry impairs the processing efficiency to a greater extent than processing effectiveness. In other words, individuals with low and high worry may show the same performance effectiveness (for example, accuracy) but exhibit a lower processing efficiency (for example, longer reaction times). Taken together, worry may negatively impact WM performance, particularly WM speed, by occupying available cognitive resources.

Recent evidence provides support for the basic assumptions of ACT [13, 14]. A number of studies found significant associations between high trait worry and decreased WM performance [15, 16]. Moreover, impairments in WM performance have been consistently observed when worry was induced [17, 18]. Although there seems to be a growing amount of evidence indicating that worry impairs WM performance, the majority of the presented studies investigated student populations with elevated levels of worry. Therefore, examining clinical levels of worry, for example in anxiety disorders such as GAD could offer further insight into questions such as “Is worry and its associated WM deficits only relevant in vulnerable populations (i.e., high worriers) when the diagnostic criteria for a mental disorder are not fulfilled or is the WM deficit associated with worry also relevant in the context of a mental disorder (e.g., GAD)?”

To date, only few studies investigated WM performance in clinical patients showing elevated worry levels [19, 20]. Whereas some studies indicate WM impairments in line with the predictions of ACT in patients with different anxiety disorders [21], other studies indicated that WM performance might not be generally disrupted in GAD patients compared to healthy controls but rather more specific aspects of WM may be impaired in anxiety disorders [19, 20]. To conclude, whereas worry seems to be associated with WM performance impairments, clinical status (e.g., GAD, another anxiety disorders) does not seem to be consistently linked with WM impairments.

The presented literature is diverse and studies differ to a great extent in regard to the WM measures, sample population, and WM task duration. For example, although a range of different tasks exist to measure WM capacity (e.g. [22]), the tasks differ in the underlying WM function they assess. Miyake and colleagues [10] proposed three separable WM functions, namely updating, inhibition and shifting. Preliminary evidence suggests that worry might particularly impair WM updating [16, 17], such as updating and removing irrelevant material from WM. However, a few studies investigated WM updating in healthy, subclinical and clinical populations. Connected to this notion and most striking, the majority of studies used student or co-worker samples and only few studies used patients with anxiety or other mental disorders. Moreover, it does not seem to be clear how much the operationalization of worry levels varied substantially: In some studies, anxiety disorder characterized by extreme worry (such as GAD) have been treated as representative for pathological worry, whereas in other studies, self-report worry measures have been used to index worry. Finally, the majority of studies used a short WM task, with a maximum of 200 trials and there is a lack of studies using WM tasks lasting longer than 10 min. Recent research using longer WM tasks indicates that participants improve their WM performance over time on task, due to learning or practice effects [23] which are defined as “an increase in a subject’s test score from one administration to the next” [24]. However, it is unclear how worry and clinical status influence the WM performance change over time in longer WM tasks [25].

### The current study

The present study investigates how worry and clinical status influence WM performance. Participants performed an extended WM task consisting of two WM blocks (Block 1 and Block 2) lasting in total around 30 min. In this study, *block* denotes a complete WM task that is usually undertaken by participants as a single task [18]. WM performance was measured as accuracy and reaction time. Two analytic strategies are applied to operationalize worry. First, worry is measured based on the self-reported worry score of each participant. Second, based on the current clinical status of each participant, four groups were considered (GAD group, clinical group with another anxiety or mood disorder; subclinical group reporting excessive worry but did not meet any disorder criteria; control group). We formulated the following hypotheses:

#### Hypothesis 1 (worry levels)

According to ACT [9], we expect individuals with high worry levels to show similar accuracy, but longer reaction times in WM Block 1 compared to individuals with low worry.

#### Hypothesis 2 (clinical status)

We expect individuals the GAD, clinical and subclinical groups to show similar accuracy, but longer reaction times in WM Block 1, compared to the control group [9].

#### Hypothesis 3 (change from block 1 to block 2)

In general, based on studies investigating learning effects in WM tasks [23], we expect an increase in accuracy and decrease in reaction time from Block 1 to Block 2. At the same time, we explore how worry levels and clinical status might affect the change in accuracy and reaction time from Block 1 to Block 2.

## Methods

### Study design

The present study was part of the recruitment phase of a randomized-controlled trial (RCT) for cognitive-behavioural therapy for GAD patients [26]. This study protocol was approved by the Ethical Committee of Zurich (BASEC 2016–00773). Using a traditional MANOVA – design with two repeated measurements on a sample of 138 participants considered in four groups, with an alpha level of 0.05 (two-tailed) and a power of 0.80, we are able to detect an effect of size *V* = 0.29 indicated by the Pillais-Bartlett-Trace (G-Power statistical software, [27]).

### Participants

The total sample included in this study consisted of 138 individuals (67% females), with age ranging from 19 to 61 years (*M* = 27.7, *SD* = 7.39). The sample was comprised of RCT participants and a control group composed of students from the University of Zurich. In the RCT sample, the Structured Clinical Interview for DSM-IV (SCID [28];) was applied to assess the current clinical status. Overall, 36 individuals met diagnostic criteria for a primary diagnosis of GAD, 33 individuals met the diagnostic criteria for a current anxiety disorder (other than GAD) or mood disorder, and 27 individuals reported excessive worries but did not meet the full criteria for a diagnosis (for a more detailed description of diagnosis and comorbid disorders, see Additional file 1).

#### Groups

Based on the current clinical status, three groups were formed: A GAD group (*n* = 36), a clinical group (*n* = 33) and a subclinical group (*n* = 27). The fourth group was the control group (*n* = 42). The four groups did not differ in sex, nationality or socio-economic status (see Supplementary Table 1, Additional File 1). However, the control group was significantly younger than the GAD group (difference = 4.31, *t* (50) = 3.23, *p =* .01) as indicated by the Kruskal- Wallis test and the Games-Howell correction. Eta square was calculated as a measure of effect size and indicates a moderate effect *η2* = 0.081.

Worry at intake, assessed with the Penn State Worry Questionnaire (PSWQ, [29]), was highest in the GAD group (*M* = 65.8, *SD* = 5.9), followed by the clinical group (*M* = 61.9, *SD* = 7.8) and the subclinical group (*M* = 59.3, *SD* = 11.1). The control group reported the lowest worry scores (*M* = 41, *SD* = 8.9). The Kruskal-Wallis test and the Games-Howell correction revealed significant group differences in worry scores (*H* [3] = 75.85, *p >* .001) and effect size calculations indicated a large effect measured by eta square (*η2* = 0.54). The control group reported significantly less worry than the GAD group (difference = 24.8, *p* = < .001), the clinical group (difference = 21, *p* = < .001) and the subclinical group (difference = 18.4, *p* = < .001). Further, the subclinical group reported significantly lower worry scores than the GAD group (difference = 6.4, *p* = .04). These results confirm the grouping of the GAD, clinical, subclinical and control individuals. Furthermore, PSWQ scores were comparable to other studies using clinical groups and control groups [30].

### Procedure

**Fig. 1:**
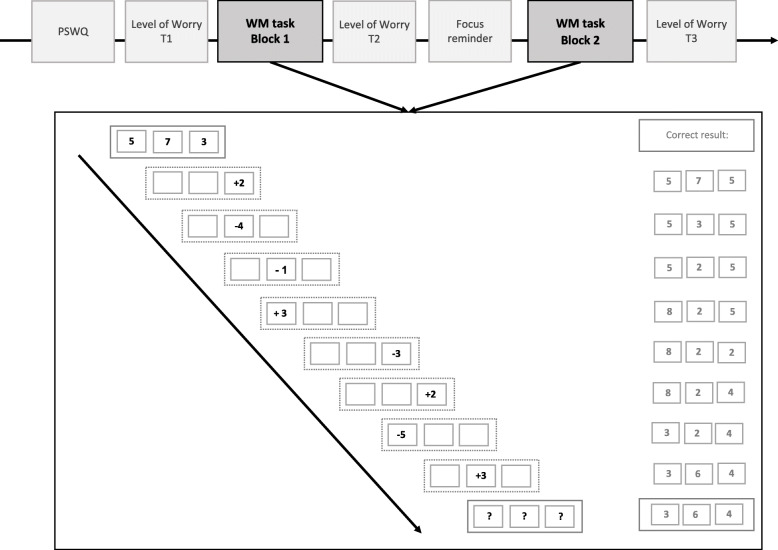
Details of experimental procedure. The example shows a trial of the working memory task with 3 set-sizes (three boxes are presented) consisting of eight updating steps in dotted squares (arithmetic operations) and a recall run (indicated by question marks at the end of the updating steps). The correct results of each updating step as well as the recall trial are presented on the right and were typed in by participants after each updating step. T1 = time 1; T2 = time 2; T3 = time 3. PSWQ = Penn State Worry Questionnaire. WM = Working memory

Participants performed two WM blocks (Block 1 and 2) and rated their current worry level at three time points (T1: before WM Block 1; T2: between WM Block 1 and 2; T3: after WM Block 2; see Fig. 1). First, in an initial warm-up phase, participants filled out the informed consent form for study participation, the PSWQ and rated their current level of worry (T1). Next, the WM task was explained verbally by the experimenter and participants completed two practice trials. Afterwards, the experimenter left the room and WM Block 1 started. After the first WM Block, participants rated their current level of worry (T2). Next, a text was presented on the screen reminding participants to focus and concentrate. This text represents a focus reminder. By clicking a key, participants started WM Block 2. After the completion of WM Block 2, participants were again asked to rate their level of worry (T3). Afterwards, the experimenter thanked participants for their participation and answered questions.

All of the participants performed the WM task under comparable conditions with an initial warm-up phase before the task started. This warm-up phase was of comparable length and intensity for all RCT and control participants. After the completion of the WM task, the RCT participants started the SCID-interview.

### Materials

#### Questionnaires

The Penn State Worry Questionnaire (PSWQ, 23) is a 16-item self-reported questionnaire assessing pathological worry. Total scores in the present study ranged from 23 to 77, with higher scores representing greater worry. Internal consistency in the present study was excellent (Cronbach’s *α* = .93).

The current level of worry was assessed with a visual analogue scale ranging from 0 to 100 (0 = no worry to 100 = extreme worry). Self-administrated measures for subjective phenomena have been proven useful in many fields [31].

#### Working memory task

The “Memory Updating task” [32] was used to assess WM performance (for a detailed task description, see Additional file 1). The WM task was administered on a 15-in. laptop using the Java-based platform Tatool [33]. In total, each WM block consisted of 150 trials. In the beginning of each WM trial, participants were presented with three or four boxes with a number in each box. In a next step, in one of the boxes, an arithmetic operation appeared and participants were asked to recall the previously presented numbers, then to perform the arithmetic operation and type in the result (“updating trial”). This was repeated eight times (eight updating trials) in which each time, an arithmetic operation appeared in one of the boxes (in the same as in the previous trial or in another box) and participants again had to recall the previously presented numbers, perform the arithmetic operation and type in the result. After eight additional arithmetic operations, question marks appeared in each box and participants were asked to type in the last number they recall (“recall trial”).

The two blocks were alternate versions of the same updating task. Thus, the general structure of the blocks was identical (for each block 42 recall trials, 108 updating trials) but the presented numbers and arithmetic operations were randomized for each block in order to avoid a possible learning effect. The WM task was self-paced and participants needed on average 15 min per WM block.

### Data analysis

The main goal of the current study was to examine how worry and the clinical status influence WM accuracy and reaction time in Block 1 as well as the change from Block 1 to Block 2 (predictor by Block interaction). A multivariate multilevel modelling approach was used to simultaneously analyse the two outcome variables (accuracy and reaction time) as well as the nested data structure (repeated measures nested in individuals). The equations for the multilevel model as well as detailed information on data preparation can be found in the Additional file 2. Multilevel modelling was performed using “multilevel” [34] and “nlme” [35] packages in R statistical software version 3.5.1 [36]. In all of the models, accuracy and reaction time were the dependent variables, the two WM blocks (Block 1, Block 2) at level-1 were nested in individuals at level-2, worry and group were entered as level 2 predictors (Model 1a (Worry), Model 2a (Group)). Furthermore, analysis of the demographic characteristics revealed a significant age difference between the groups and therefore, age was included as a covariate in both models (Model 1b (Worry + age), Model 2b (Group + age)).

## Results

Descriptive statistics on the total sample indicate a mean accuracy of 87.6 (*SD* = 11.4) in Block 1 which decreased to 81.8 (*SD* = 9.4) in Block 2. In addition, mean reaction time across all participants decreased from 2581 ms (*SD* = 652 ms) in Block 1 to 2451 ms (*SD* = 611) in Block 2 (for further descriptive and inferential statistical information as well as effect size calculations, see Supplementary Tables 2 to Table 6, Additional File 3). With regard to the level of worry assessed with a visual analogue scale at three time points, results indicated that at T1, the GAD, clinical, and subclinical group showed significantly higher worry levels compared to the control group (subclinical: *t* (134) = 3.31, *p* = .002; clinical: *t* (134) = 6.02, *p* > .000; GAD: *t* (134) = 4.67, *p* > .000). Further, in the control group, worry decreased significantly from T1 to T2 (*t* (268) = − 2.4, *p* = .016) and from T1 to T3 (*t* (268) = − 2.7, *p* = .006). There was no significant Group by Block interaction.

### Hypothesis 1 (worry levels)

**Fig. 2:**
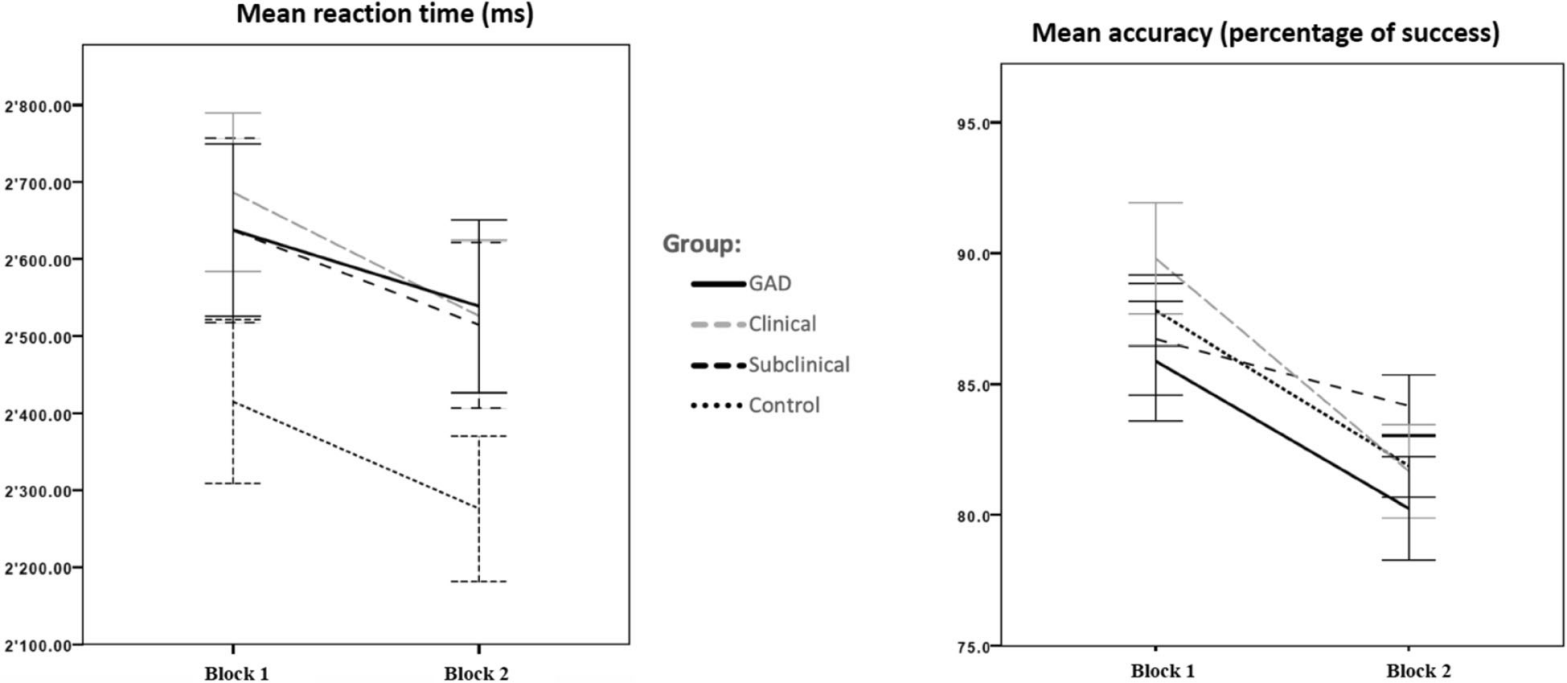
Mean Accuracy and reaction time for the GAD, clinical, subclinical and control group in Block 1 and Block 2. Accuracy values in percentage of successful trials; Reaction time in milliseconds (ms); Control = Control group; Subclinical = Subclinical group; Clinical = Clinical group; GAD = Generalized anxiety disorder group; Error bars represent standard errors

In Block 1, there was no significant main effect of worry on accuracy (*t* (407) = − 1.3, *p* = .19; Model 1a). However, higher worry scores were significantly associated with longer reaction times in Block 1 (*t* (407) = 2.41, *p* = .01), Fig. 2). When age was included (Model 1b), the results again indicated no significant main effect of worry on accuracy (*t* (396) = − 1.43, *p* = .15). Again, a significant main effect of worry on reaction time was obtained (*t* (396) = 2.46, *p* = .01) in Block 1. There was no significant main effect of age on accuracy (*t* (396) = − 0.66, *p* = .50) or reaction time (*t* (396) = −.018, *p* = .85).

### Hypothesis 2 (groups)

On a descriptive level, mean accuracy in Block 1 was highest in the clinical group (*M* = 89.8, *SD* = 12.3), followed by the control group (*M* = 87.8, *SD* = 8.7), the subclinical group (*M* = 86.7, *SD* = 11.1) and the GAD group (*M* = 85.8, *SD* = 13.6). For reaction time, in Block 1, the control group was the fastest group (*M* = 2415 ms, *SD* = 689), followed by the subclinical (*M* = 2637 ms, *SD* = 627), and GAD group (*M* = 2637 ms, *SD* = 647 ms), with the clinical group being the slowest group (*M* = 2686 ms, *SD* = 589). When performing the multivariate multilevel model (Model 2a), there were no significant differences in accuracy and reaction time between the control group and all of the other groups in Block 1. When age was included (Model 2b), accuracy mean scores did not differ between the control group and all of the other groups in Block 1. However, the GAD, clinical and subclinical group showed significantly longer reaction times in Block 1, compared to the control group (GAD: *t* (380) = 2.15, *p* = .03; clinical: *t* (380) = 2.55, *p* = .01; subclinical: *t* (380) = 2.08, *p* = .03). No main effects of age on accuracy (*t* (380) = 0.92, *p* = .35) or reaction time (*t* (380) = − 1.92, *p* = .055) were obtained.

### Hypothesis 3 (change from block 1 to block 2)

When comparing WM performance between Block 1 and Block 2, the participants decreased significantly in accuracy (*t* (407) = − 8.54, *p* < .000) as well as in reaction time (*t* (407) = − 4.50, *p* < .000), regardless of worry levels. There was no significant Block by worry interaction for accuracy or reaction time. When age was included, results again indicated a significant decrease in accuracy (*t* (396) = − 8.49, *p* < .000) as well as in reaction time (*t* (396) = − 4.40, *p* < .000) across all participants. A significant worry by age interaction was obtained for accuracy (*t* (396) = − 2.08, *p* = 0.03) indicating that with higher age, the more negative the effect of worry on accuracy. No further interaction reached statistical significance.

Second, potential group differences in the change in accuracy and reaction time from Block 1 to Block 2 were explored (Group by Block interactions). From Block 1 to Block 2, the control group showed a significant decrease in accuracy (*t* (399) = − 4.99, *p* < .001) as well as in reaction time (*t* (399) = − 2.62, *p =* .009). Next, for each group (GAD, clinical and subclinical group), the change in accuracy and reaction time from Block 1 to Block 2 was computed and this change was compared to the change of the control group’s change in accuracy and reaction time from Block 1 to Block 2 (“Group by Block interaction”). No significant Group by Block interactions for accuracy or reaction time for the GAD, clinical and subclinical group compared to the control group were obtained, indicating that the rate of change in accuracy and reaction time from Block 1 to Block 2 did not differ between the groups. When age was controlled for, the control group showed a significant decrease in accuracy (*t* (380) = − 4.09, *p* = < .001) but not in reaction time (*t* (399) = − 1.09, *p =* .27) from Block 1 to Block 2. No further interaction reached statistical significance.

## Discussion

The aim of the present study was to investigate how worry and the clinical status influence working memory (WM) performance measured by accuracy and reaction time. Based on the Attentional Control Theory (ACT; 9), we expected individuals with high worry (such as the generalized anxiety disorder [GAD], clinical and subclinical group) to show similar performance effectiveness (accuracy), but longer reaction times in WM Block 1 (reduced efficiency), compared to those with low worry (such as the control group) (Hypothesis 1 & 2). Indeed, our results supported Hypothesis 1 and partly supported Hypothesis 2. In line with ACT, we found that self-reported worry levels were significantly associated with longer reaction times in WM Block 1 but did not affect accuracy.

The obtained results support existing research indicating that high trait worry impairs WM performance [15, 16]. When taking potential age effects into account, the GAD, clinical and subclinical groups showed significantly longer reaction times compared to the control group in Block 1 (Hypothesis 2). One possible explanation of this result is that when age was maintained constant, the effects of extreme worry on a (sub) clinical level became evident in the GAD, clinical and subclinical groups regardless of having a primary diagnosis or not. This suggests that worry as transdiagnostic characteristic is not only relevant in anxiety disorders but also in individuals with other subclinical or clinical psychological symptoms as well as in healthy individuals [5, 37]. However, in order to be able to draw more definitive conclusions, replication across clinical and subclinical samples with a variety of anxiety and mood disorders using carefully selected larger samples is needed. This way, the underlying thinking processes of worry and its effects on WM performance could be further investigated.

This study may provide first insight into to the question if worry and its associated WM deficits are only relevant in the context of a mental disorder (e.g., GAD) or whether worry generally affects WM processing in vulnerable populations (i.e. high worriers) even when the diagnostic criteria for a mental disorder are not fulfilled. We obtained preliminary evidence that worry is generally affecting WM performance in individuals with high worry levels, and that WM deficits associated with worry are not only relevant in the context of a mental disorder. The results are in line within the current Research Domain Criteria era which proposes that disrupted WM processes are thought to play an important role in the maintenance of pathological worry [6]. More specifically, we found higher worry to be associated with deficits in WM updating. These results are in line with a recent meta-analysis [38] which investigated the association between transdiagnostic worry and cognitive functions and results indicated an association between worry and only one specific WM function, namely difficulty in updating and discarding no longer relevant material from WM (*r* = − 0.20). Therefore, future research is needed to further investigate if particular functions of WM (i.e. updating function) are more severely impaired by worry than others WM functions [10]. To conclude, we found preliminary evidence that higher worry impairs WM updating in healthy, subclinical and clinical populations. These findings offer support for a transdiagnostic approach to psychopathology which postulates that there are common pathological thinking processes, such as worry shared across psychological disorders rather than specific and distinct processes within each disorder category [7, 39]. Moreover, the results that higher worry impairs WM updating not only in clinical, but also in subclinical and even in healthy populations indicating that the impairment in WM updating as a result of high worrying is not only specific to individuals with a clinical diagnosis, and that high worry might also affect healthy individuals` WM.

In this study we used a two-block WM task consisting of more than 300 trials [6] and the advantage of using this extended two-block task over the standard use of one block is the ability to examine the stability of accuracy and reaction over time (Hypothesis 3). Overall, all of the groups showed a similar decrease in accuracy and reaction time across the WM assessment. Moreover, regardless of worry level, participants showed a decline in accuracy and a decrease in reaction time. In other words, over time, participants became faster, but also made more errors regardless of worry level or clinical status. One possible explanation for this finding might be the nature of the WM task itself. The WM task was self-paced, meaning that the participants were able to control the timing of the stimulus presentation on their own – when they press a button, the task continued [40]. In these self-paced paradigms, the response time plays a major role [41]. Although the participants were instructed to respond as quickly as possible, it is possible that the participants took their time to perform the task as accurately as they could instead of responding at high speed. Supporting this idea, all of the groups showed high accuracy scores with no average group score below 80% successful blocks. Taken together, in order to deepen the knowledge of the change over time in WM performance, longer WM tasks with more than two blocks could provide answers concerning the nature of change in WM performance and worry [19].

There are several limitations that must be considered when interpreting the results of the present study. First, the GAD, clinical, and subclinical groups were recruited in the context of the same RCT and therefore a particular population of help-seeking individuals might have been recruited. Therefore, in order to further investigate the dimensional nature of worry, it would be helpful to include more heterogeneous transdiagnostic samples recruited from the general population. Second, this study contrasted different clinical and non-clinical samples recruited from different populations. Although this is a common procedure in such designs [19, 21, 42], the results of this study are preliminary in nature and need careful replication. Finally, we did not conduct a standardised diagnostic interview in our control group and therefore, we were not able to determine if the participants were suffering from subclinical symptoms of a mental disorder. However, the mean score of the Penn State Worry Questionnaire of the control group are similar to other control groups used in comparable studies [30, 43]. Nevertheless, in the future, more carefully designed diagnostic assessments are needed.

## Conclusion

This is one of the first studies to investigate the relationship between different worry levels and WM performance across clinical and non-clinical samples using a two-block WM task. The results are in line with ACT [9] indicating that higher worry levels negatively impact WM processing efficiency. Moreover, when age was controlled for, we found participants` clinical status to be linked with WM impairments. Future studies should aim to replicate the present results in populations with different worry levels and mental disorders.

## Supplementary information

**Additional file 1:** Participants (demographic characteristics) and detailed description of the working memory task. **Table S1.** Demographic information on the total sample and each group according to the current clinical status.

**Additional file 2.** Data preparation and statistical models.

**Additional file 3:** Descriptive and inferential statistics (including tables and figures). **Table S2.** Descriptive statistics for accuracy and reaction time **Table S3.** Descriptive statistic of the perceived level of worry. **Table S4.** Results of the Multivariate multilevel models 1a and 2a predicting accuracy and reaction time **Table S5.** Results of the Multivariate multilevel models 1b and 2b predicting accuracy and reaction time **Table S6.** Proportional reduction of explained variance represented by Pseudo-R2 for accuracy and reaction time for each model comparison **Fig. 3.** Mean level of self-reported worry for the GAD, clinical, subclinical and control group over the course of the WM task.

## Data Availability

The datasets used and/or analysed during the current study are available from the corresponding author on reasonable request.

## Abbreviations

ACT: Attentional Control Theory
GAD: Generalized anxiety disorder
PSWQ: Penn State Worry Questionnaire
RCT: Randomized controlled trial
SCID: Structured Clinical Interview for DSM-IV
WM: Working memory
