# Supplementary material for: How do worry and clinical status impact working memory performance? An experimental investigation
Source: BMC Psychiatry. 2020 Jun 19;20:317. doi: 10.1186/s12888-020-02694-x (PMC7304094; doi:10.1186/s12888-020-02694-x)
Supplement: Supplementary file 1 — Additional file 1: Participants (demographic characteristics) and detailed description of the working memory task. Table S1. Demographic information on the total sample and each group according to the current clinical status. [file 12888_2020_2694_MOESM1_ESM.doc]

**Additional file 1**

**Participants**

The present study was part of the recruitment phase of a randomized-controlled trial (RCT) for cognitive-behavioural therapy for GAD patients (1). The sample was comprised of RCT participants and a control group composed of students from the University of XXX (Table 1). The RCT participants were individuals who were invited for a diagnostic interview in the recruitment phase of an RCT following the inclusion and exclusion trial criteria (for a detailed description of the inclusion and exclusion criteria see (1)). In the RCT sample, the Structured Clinical Interview for DSM-IV (SCID; 2) was applied to assess current clinical diagnosis. Overall, 36 individuals met diagnostic criteria for a primary diagnosis of GAD, 12 of whom had a further current comorbid disorder (panic disorder *n* = 5; current MDE *n* = 2). A total of 33 individuals met the diagnostic criteria for a current anxiety (other than GAD) or mood disorder, with the majority suffering from panic disorder (*n* = 12), current major depression (*n* = 5), adjustment disorder (*n* = 4), and dysthymia (*n* = 4), agoraphobia (n = 3), specific phobia (n = 3), social phobia (n = 1) and hypochondriasis (n = 1). Four had a further current comorbid disorder (*n* = 2 social phobia, *n* = 1 panic disorder, *n* = 1 dysthymia). Finally, 27 individuals reported excessive worries amongst other symptoms but did not meet the full criteria for a diagnosis. In total, five individuals of the RCT patients indicated taking psychotropic medication on a stable basis for at least three months (*n* = 3 antidepressant; *n* = 1 benzodiazepine, *n* = 1 Methylphenidate).

**Table 1.**

*Demographic information on the total sample and each group according to the current clinical status*

|  | | **Total**  **Sample**  (*n* = 138) | **Group** | | | |
| --- | --- | --- | --- | --- | --- | --- |
|  | |  | GAD  (*n* = 36) | Clinical  (*n* = 33) | Subclinical  (*n* = 27) | Control  (*n* = 42) |
|  | |  |  |  |  |  |
|  | |  |  |  |  |  |
| **Age** | | 27.7 (7.4) | 29.2 (7.2) | 29.3 (8.9) | 28.1 (8.7) | 24.9 (3.7) |
| **Sex** | |  | | | | |
|  | male | 32.6% | 27.8% | 30.3% | 40.7% | 33.3% |
| female | | 67.4% | 72.2% | 69.7% | 59.3% | 66.7% |
|  | |  |  |  |  |  |
| **Nationality** | |  | | | | |
|  | CH | 68.1% | 58.3% | 69.7% | 74.1% | 71.4% |
|  | DE | 17.4% | 25% | 12.2% | 14.8% | 16.7% |
| Other | | 12.3% | 16.7% | 9.1% | 11.1% | 11.9% |
|  | |  |  |  |  |  |
| **SES** | | 6.7 (1.4) | 6.9 (1.11) | 6.2 (1.6) | 6.5 (1.1) | 6.8 (1.7) |
|  | |  |  |  |  |  |
| **Symptom measures** | |  |  |  |  |  |
| PSWQ | | 56 (13.25) | 65.8 (5.9) | 61.9 (7.8) | 59.3 (11.1) | 41 (8.9) |
|  | |  |  |  |  |  |

*Note.* Values for age, SES, and PSWQ in mean (standard deviation). Frequency values for sex and nationality in percentage. GAD = generalized anxiety disorder; Nationality: CH = Switzerland, DE = Germany, Other = other nationalities; SES = Socio-economic status; scores ranging from 1 (“very low) to 10 (“very high”); PSWQ = Penn State Worry Questionnaire.

**Materials**

**Adaption of the working memory task.** The feasibility of the verbal working

memory (WM) task called “Memory Updating task” (3) was investigated in a preliminary testing with a small subgroup of patients during the recruitment phase of the randomized controlled trial (*n* = 3; 1). In the original task, three to five boxes (called “set size”, representing the level of difficulty) were presented on the screen, with numbers shown in the boxes. Participants were asked to remember the presented number in each box and to perform subsequent arithmetic operations. In total, 312 trials in each block were performed. The preliminary testing with the patients revealed that participants needed on average twice as long as usual for the task (50- 70 minutes) and they reported high frustration, insecurity and a too high level of difficulty of the task. Therefore, the task was adapted for the RCT population in order to prevent potential drop-out and exhaustion. The set size 5 (trials with five boxes) was removed, therefore the task became less difficult and shorter.

References

1. Flückiger C, Wolfer C, Held J, Hilpert P, Rubel J, Allemand M, et al. How to customize a bona fide psychotherapy for generalized anxiety disorder? A two-arms, patient blinded, ABAB crossed-therapist randomized clinical implementation trial design [IMPLEMENT 2.0]. BMC Psychiatry. 2018;18(1).

2. Wittchen HU, Zaudig M, Fydrich T. Strukturiertes Klinisches Interview für DSM-IV. Achse I und II. Handlungsanweisung. Göttingen: Hogrefe; 1997.

3. Oberauer K, Süß HM, Schulze R, Wilhelm O, Wittmann WW. Working memory capacity - Facets of a cognitive ability construct. Pers Individ Dif. 2000;29(6):1017–45.
