# Supplementary material for: How do worry and clinical status impact working memory performance? An experimental investigation
Source: BMC Psychiatry. 2020 Jun 19;20:317. doi: 10.1186/s12888-020-02694-x (PMC7304094; doi:10.1186/s12888-020-02694-x)
Supplement: Supplementary file 2 — Additional file 2. Data preparation and statistical models. [file 12888_2020_2694_MOESM2_ESM.doc]

**Additional file 2**

**Data preparation**

Prior to the data analysis, reaction time outliers were defined as reaction time data points exceeding the individual mean of each participant more than three standard deviations and were excluded (1, 2). Following this rule, approximately 2% of the data of Block 1 and Block 2 were removed. Further, only reaction times of correct responses were included in the analysis. In line with the recommendations of Osborne and Overbay (2), extreme accuracy data defined as exceeding the RCT and control sample mean by more than three standard deviations was excluded (control group: *n* = 1, RCT sample: *n* = 2). In these three cases, accuracy was so low that we assumed that the participants did not understand or comply with the task instructions.

Accuracy and reaction time were assessed repeatedly in two blocks (Blocks 1 and 2) and analysed as outcome variables. Due to the likeliness that the two outcomes of the same person were related, multilevel modelling was performed using “multilevel” (3) and “nlme” (4) packages in R statistical software (5). To investigate how worry and the clinical status indexed by the four groups influence accuracy and reaction time in Block 1 as well as the change from Block 1 to Block 2, a series of two-level multivariate analyses were conducted. In all of the models, the accuracy and RT were the dependent variables, the two WM blocks (WM Block 1 and Block 2) at level-1 were nested in individuals at level-2 (6,7).

**Model 1a (Worry).** In this model, the accuracy and reaction time were the dependent variables, the two WM blocks (WM Block 1 and Block 2) were nested at level-1 nested in individuals at level-2 and the PSWQ score was grand-mean centered (as a measure of worry) and entered as a level-2 characteristic in the model.

**Model 1b (Worry + age).** In order to control for potential age effects, age was grand-mean centered and included in Model 1a as a covariate.

**Model 2a (Group).** The four groups (GAD group, clinical group, subclinical group, and control group) were treated as an individual characteristic and group was considered as a level-2 predictor. The four groups were dummy coded, with the control group as the reference group. Block was coded with Block 1 = 0 and Block 2 = 1, with Block 1 representing the baseline accuracy and reaction time measure.

**Model 2b (Group + age)**. In order to control for potential age effects, age was grand-mean centered and added into Model 2a as a covariate.

**Model equation**

**Model 2a (Group).** For the multivariate mixed effect analysis of Model 2a, with accuracy and reaction time as dependent variables and Block and Group as predictors, an indicator variable *h* was created indicating the type of response being analysed where *h* = 1 for accuracy (a_j_) and *h* = 2 for reaction time (rt_j_). For each outcome, the following univariate mixed model was defined:

| The value Y_hij_ for the *accuracy* outcome is: | |
| --- | --- |
| (1) | (Y_1ij_) = β_10_ + β_11_ (Block_ij_) + β_12-14_ (Group_j_) + β_15-17_ (Block_ij_ * Group_j_) + μ_1j_ + r_ij_ |
| The value Y_hij_ for the *reaction time* outcome is: | |
| (2) | (Y_2ij_) = β_20_ + β_21_ (Block_ij_) + β_22-24_ (Group_j_) + β_25-27_ (Block_ij_ * Group_j_) + μ_2j_ + r_ij_ |

Focusing on Equation (1), the indicator *h* for accuracy (*h*=1) at time i for person j is a function of the overall intercept (β_10_) which is equal to the expected mean value of accuracy when all predictors are zero (in this case when Block = 0 (Block 1) and Group = 0 (Control group)), the slope of accuracy (β_11_), representing the average rate of change in accuracy from Block 1 to Block 2 for the Control group, a coefficient for each sample (β_12-14_) indicating the mean difference between Control group compared to the GAD, Clinical and Subclinical group in accuracy score at baseline (Block = 0), the interaction term (β_15-17_) for each Block by Group interaction, indicating the differences in rate of change of the Control group compared to the rate of change of the GAD, Clinical and Subclinical group and a random effects: μ_1j_ representing person-specific differences at block1 (unique baseline values for each participant) and r_ij_, the residual error. Interpretation of the reaction time outcome in Equation (2) is identical to accuracy outcome, with the corresponding reaction time coefficients.

**Model 1a.** For the multivariate mixed effect analysis of Model 2a, with accuracy and reaction time as dependent variables and Block and grand-mean centered worry score (measured with the Penn State Worry Questionnaire; 8) as predictors, the same equation and interpretation as presented in Model 2a applies.

References

1. Miller J. Reaction time analysis with outlier exclusion- Bias varies with sample size. Q J Exp Psychol. 1991;43(3):907–12.

2. Osborne JW, Overbay A. The power of outliers (and why researchers should always check for them). Pract Assessment, Res Eval. 2004;9(6).

3. Bliese P. multilevel: Multilevel Functions. 2016.

4. Pinheiro J, Bates D, DebRoy S, Sarkar D, Team RC. nlme: Linear and Nonlinear Mixed Effects Models [Internet]. 2018. Available from: https://cran.r-project.org/package=nlme

5. R Core Team. R: A language and environment for statistical computing. Vienna, Austria: R Foundation for Statistical Computing; 2018.

6. Baldwin SA, Imel ZE, Braithwaite SR, Atkins DC. Analyzing Multiple Outcomes in Clinical Research Using Multivariate Multilevel Models. J Consult Clin Psychol. 2014;82(5):920–30.

7. Snijders TA, Boskers RJ. Multilevel Analysis - An Introduction to Basic and Advanced Multilevel Modelling. 2nd ed. London: Sage Publisher; 2012.

8. Stoeber J. Reliability and validity of two widely-used worry questionnaires: self-report and self-peer convergence. Pers Individ Dif [Internet]. 1998;24(6):887–90. Available from: http://dx.doi.org/10.1016/S0191-8869(97)00232-8
