# Supplementary material for: How do worry and clinical status impact working memory performance? An experimental investigation
Source: BMC Psychiatry. 2020 Jun 19;20:317. doi: 10.1186/s12888-020-02694-x (PMC7304094; doi:10.1186/s12888-020-02694-x)
Supplement: Supplementary file 3 — Additional file 3: Descriptive and inferential statistics (including tables and figures). Table S2. Descriptive statistics for accuracy and reaction time Table S3. Descriptive statistic of the perceived level of worry. Table S4. Results of the Multivariate multilevel models 1a and 2a predicting accuracy and reaction time Table S5. Results of the Multivariate multilevel models 1b and 2b predicting accuracy and reaction time Table S6. Proportional reduction of explained variance represented by Pseudo-R2 for accuracy and reaction time for each model comparison Fig. 3. Mean level of self-reported worry for the GAD, clinical, subclinical and control group over the course of the WM task. [file 12888_2020_2694_MOESM3_ESM.docx]

**Additional file 3**

**Table 2.**

*Descriptive statistics for accuracy and reaction time*

|  |  | **Accuracy** | | |  | **Reaction time** | | |
| --- | --- | --- | --- | --- | --- | --- | --- | --- |
|  |  | Block 1 | Block 2 | Total |  | Block 1 | Block 2 | Total |
|  |  | *M (SD)* | *M (SD)* | *M (SD)* |  | *M (SD)* | *M (SD)* | *M (SD)* |
|  |  |  |  |  |  |  |  |  |
| **Group:** |  |  |  |  |  |  |  |  |
| GAD |  | 85.8 (13.6) | 80.2 (11.9) | 83.1 (13) |  | 2637 (647) | 2539 (669) | 2588 (669) |
| Clinical |  | 89.8 (12.3) | 81.7 (10.2) | 85.7 (11.9) |  | 2686 (589) | 2526(565) | 2606 (578) |
| Subclinical |  | 86.7 (11.1) | 84.2 (6.1) | 85.4 (8.9) |  | 2637 (627) | 2514(559) | 2575 (589) |
| Control |  | 87.8 (8.7) | 81.9 (7.8) | 84.8 (8.7) |  | 2415 (689) | 2276(611) | 2345 (651) |
| **Total** |  | 87.6 (11.4) | 81.8(9.4) | 84.7 (10.8) |  | 2581 (652) | 2451(611) | 2516 (634) |
|  |  |  |  |  |  |  |  |  |

*Note.* Accuracy is given in percentage of correct responses; reaction time is given in milliseconds; M = mean; SD = standard deviation; GAD = Generalized anxiety disorder.

**Table 3.**

*Descriptive statistic of the perceived level of worry.*

|  |  | **Worry level** | | | | | | | |
| --- | --- | --- | --- | --- | --- | --- | --- | --- | --- |
|  |  | T1 | |  | T2 | |  | T3 | |
|  |  | *M* | *(SD)* |  | *M* | *(SD)* |  | *M* | *(SD)* |
| **Group:** |  |  |  |  |  |  |  |  |  |
| GAD |  | 42.5 | (21.6) |  | 40.2 | (23.5) |  | 40.76 | (22.5) |
| Clinical |  | 49.1 | (21.9) |  | 37.06 | (20.3) |  | 38.49 | (20.9) |
| Subclinical |  | 36.9 | (19.3) |  | 29.5 | (17.7) |  | 31.5 | (19.6) |
| Control |  | 21.81 | (14.9) |  | 16.34 | (11.5) |  | 15.5 | (12) |
|  |  |  |  |  |  |  |  |  |  |

*Note.* Worry scores are presented in absolute scores of the visual analogue scale (range 0 to 100). T1 = time 1 (before working memory Block 1); T2 = time 2 (between working memory Block 1 and 2); T3 = time 3 (after working memory Block 2); M = mean; SD = standard deviation; GAD = Generalized anxiety disorder.

Fig. 3.

**Mean level of self-reported worry for the GAD, clinical, subclinical and control group over the course of the WM task.**


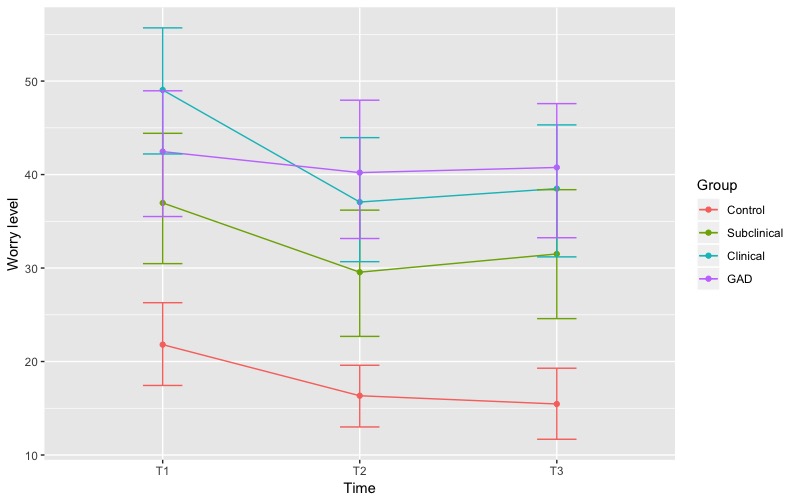


*Note.* Worry levels were assessed with a visual analogue scale ranging from 0 to 100. T1 = time 1; T2 = time 2; T3 = time 3.

**Table 4.**

*Results of the Multivariate multilevel models 1a and 2a predicting accuracy and reaction time*

| **Fixed effects** |  | **Accuracy** | | |  | **Reaction time** | | | |
| --- | --- | --- | --- | --- | --- | --- | --- | --- | --- |
|  |  | **Model 1a (Worry)** |  | **Model 2a (Group)** |  | **Model 1a (Worry)** |  | **Model 2a (Group)** |  |
|  |  |  |  |  |  |  |  |  |  |
| intercept |  | 87. 56 (0.88) *** |  | 87.8 (1.61) ***** |  | 2581.52 (52.6) *** |  | 2415.08 (97.11) ***** |  |
| Block |  | -5.72 (0.67) *** |  | - 5.93 (1.18) ***** |  | -130.6 (28.98) *** |  | -139.10 (52.96) **** |  |
|  |  |  |  |  |  |  |  |  |  |
| **Group:** |  |  |  |  |  |  |  |  |  |
| GAD |  |  |  | -1.92 (2.38) |  |  |  | 222.69 (142.94) |  |
| Clinical |  |  |  | 1.98 (2.43) |  |  |  | 271.42 (146.40) |  |
| Subclinical |  |  |  | -1.09 (2.58) |  |  |  | 222.03 (155.08) |  |
|  |  |  |  |  |  |  |  |  |  |
| **Worry** |  | -0.08 (0.06) |  |  |  | 9.64 (3.98) * |  |  |  |
|  |  |  |  |  |  |  |  |  |  |
| **Block * Group:** |  |  |  |  |  |  |  |  |  |
| Block * GAD |  |  |  | 0.28 (1.75) |  |  |  | 40.04 (77.97) |  |
| Block * Clinical |  |  |  | -2.19 (1.79) |  |  |  | -21.49 (79.82) |  |
| Block * Subclinical |  |  |  | 3.39 (1.90) |  |  |  | 16.30 (84.68) |  |
|  |  |  |  |  |  |  |  |  |  |
| **Block * Worry** |  | 0.01 (0.05) |  |  |  | 2.0 (2.19) |  |  |  |
|  |  |  |  |  |  |  |  |  |  |

| **Random effects** | **Accuracy** | |  | **Reaction time** | | |
| --- | --- | --- | --- | --- | --- | --- |
|  | Variance | SD |  | Variance | SD | |
| **Model 1a (Worry)** |  |  |  |  |  |  |
| Intercept | 76.91 | 8.77 |  | 319718.96 |  | 565.43 |
| Residual | 30.56 | 5.52 |  |  |  |  |
| **Model 2a (Group)** |  |  |  |  |  | |
| Intercept | 77.76 | 8.81 |  | 326577.54 | 571.46 | |
| Residual | 28.87 | 5.37 |  |  |  |  |
|  |  |  |  |  |  |  |

*Note*. Coefficients (standard error) are presented for the fixed effects*.* Model 1a: Working memory Block as a level-1 predictor, worry as a level-2 predictor; Model 2a: Working memory Block as a level-1 predictor, Group as level-2 predictor; SD = standard deviation; Random effects of the intercept of each outcome were estimated.

**p* < .05. ** *p* < .01. ****p* < .001.

**Table 5.**

*Results of the Multivariate multilevel models 1b and 2b predicting accuracy and reaction time*

| **Fixed effects** |  | **Accuracy** | | |  | **Reaction time** | | |
| --- | --- | --- | --- | --- | --- | --- | --- | --- |
|  |  | **Model 1b (Worry + age)** |  | **Model 2b**  **(Group + age)** |  | **Model 1b**  **(Worry + age)** |  | **Model 2b**  **(Group + age)** |
|  |  |  |  |  |  |  |  |  |
| intercept |  | 87.69 (0.89) *** |  | 88.7 (2.28) *** |  | 2578.77 (53.36) *** |  | 2270.12 (129.37) *** |
| Block |  | -5.78 (0.68) *** |  | - 6.38 (1.55) *** |  | -129.60 (29.44) *** |  | -74.69 (68.18) |
|  |  |  |  |  |  |  |  |  |
| **Group:** |  |  |  |  |  |  |  |  |
| GAD |  |  |  | -2.3 (2.75) |  |  |  | 356.94 (165.42) * |
| Clinical |  |  |  | 0.97 (2.79) |  |  |  | 429.47 (168.19) * |
| Subclinical |  |  |  | -2.17 (2.91) |  |  |  | 365.57 (175.04) * |
|  |  |  |  |  |  |  |  |  |
| **Worry** |  | -0.09 (0.06) |  |  |  | 10.02 (4.06) * |  |  |
|  |  |  |  |  |  |  |  |  |
| **Age** |  | -0.08 (0.12) |  | 0.41 (0.45) |  | -1.33 (7.26) |  | -52.36 (27.24) |
|  |  |  |  |  |  |  |  |  |
| **Block * Group:** |  |  |  |  |  |  |  |  |
| Block * GAD |  |  |  | 0.57 (2.05) |  |  |  | - 13.52 (89.80) |
| Block * Clinical |  |  |  | -1.73 (2.08) |  |  |  | -86.14 (91.30) |
| Block * Subclinical |  |  |  | 3.83 (2.16) |  |  |  | - 43.44 (95.0) |
|  |  |  |  |  |  |  |  |  |
| **Block * Worry** |  | 0.02 (0.05) |  |  |  | 2.08 (2.24) |  |  |
|  |  |  |  |  |  |  |  |  |
| **Block * Age** |  | 0.012 (0.09) |  | -0.14 (0.33) |  | -3.20 (4.0) |  | 21.61 (14.79) |
|  |  |  |  |  |  |  |  |  |
| **Group * Age** |  |  |  |  |  |  |  |  |
| GAD * Age |  |  |  | - 0.87 (0.51) |  |  |  | 59.66 (31.0) |
| Clinical * Age |  |  |  | - 0.44 (0.49) |  |  |  | 44.02 (29.95) |
| Subclinical * Age |  |  |  | - 0.36 (0.55) |  |  |  | 55.52 (30.73) |
|  |  |  |  |  |  |  |  |  |
| **Age * Worry** |  | -0.01 (0.008) * |  |  |  | 0.50 (0.53) |  |  |
|  |  |  |  |  |  |  |  |  |
| **Block * Group * Age** |  |  |  |  |  |  |  |  |
| Block * GAD * Age |  |  |  | 0.25 (0.38) |  |  |  | - 29 (16.83) |
| Block * Clinical * Age |  |  |  | 0.13 (0.37) |  |  |  | - 21.45 (16.25) |
| Block * Subclinical * Age |  |  |  | 0.15 (0.38) |  |  |  | -32.01 (16.68) |
|  |  |  |  |  |  |  |  |  |
| **Block * Worry * Age** |  | 0.005 (0.006) |  |  |  | -0.03 (0.29) |  |  |
|  |  |  |  |  |  |  |  |  |

| **Random effects** | **Accuracy** | |  | **Reaction time** | | |
| --- | --- | --- | --- | --- | --- | --- |
|  | Variance | SD |  | Variance | SD | |
| **Model 1b (Worry + age)** |  |  |  |  |  |  |
| Intercept | 74.64 | 8.63 |  | 318666.64 |  | 564.50 |
| Residual | 30.58 | 5.53 |  |  |  |  |
|  |  |  |  |  |  |  |
| **Model 2b (Group + age)** |  |  |  |  |  | |
| Intercept | 75.38 | 8.68 |  | 321313.92 | 566.84 | |
|  |  |  |  |  |  |  |

*Note*. Coefficients and standard error in parentheses are presented for the fixed effects*.* Model 1b: Working memory Block as a level-1 predictor, PSWQ and Age as level-2 predictors; Model 2b: Working memory Block as a level-1 predictor, Group and Age as level-2 predictors; SD = standard deviation; Random effects of the intercept of each outcome were estimated. PSWQ = Penn State Worry Questionnaire.

**p* < .05. ** *p* < .01. ****p* < .001.

**Effect sizes**

In order to estimate the effect sizes for the different models, we calculated Peusdo-R^2^ values representing the proportion of explained variance by the predictor variable. More specifically, Pseudo-R^2^ represents the proportional reduction in random intercept variance of the conditional models in comparison to the unconditional model without predictor(s) (1). In all models, accuracy and reaction time were the dependent variables, the two WM blocks (WM Block 1 and Block 2) at level-1 were nested in individuals at level-2. We calculated an empty model with Block as a predictor (Model 0) representing the unconditional model. Block was coded with Block 1 as 0 and Block 2 as 1, with Block 1 representing the baseline WM performance measure. Pseudo-R^2^ is calculated for the following models. Results obtained for Model 0 indicate a mean accuracy of 87.5% and a mean reaction time of 2581 ms across all participants in Block 1. From Block 1 to Block 2, participants significantly decreased in accuracy (*t* (411) = -8.56, *p >* .001) as well as in reaction time (*t* (411) = -4.5, *p >* .001).

In the next step, we calculated the proportional reduction of explained variance represented by Pseudo-R^2^ for each predictor added to the model. The results are displayed in Table 6.

**Table 6.**

*Proportional reduction of explained variance represented by Pseudo-R^2^ for accuracy and reaction time for each model comparison*

| **Model name** |  | **Predictors** | **Model comparisons** |  | **Peusdo-R^2^** | | |
| --- | --- | --- | --- | --- | --- | --- | --- |
|  |  |  |  |  | Accuracy |  | Reaction time |
| Model 0 |  | Block |  |  |  |  |  |
| Model 1a |  | Block, Worry | Model 0 vs.  Model 1a |  | 0.0136 |  | 0.0577 |
| Model 2a |  | Block, Group | Model 0 vs.  Model 2a |  | 0.0028 |  | 0.0375 |
| Model 1b |  | Block, Worry, Age | Model 1a vs.  Model 1b |  | 0.0295 |  | 0.0032 |
| Model 2b |  | Block, Group, Age | Model 2a vs.  Model 2b |  | 0.0305 |  | 0.016 |

*Note.* Pseudo-R^2^ represents the proportional reduction of intercept variance. In all models, accuracy and reaction time were dependent variables and pseudo-R^2^ is calculated separately for each dependent variable.

References

1. Baldwin SA, Imel ZE, Braithwaite SR, Atkins DC. Analyzing Multiple Outcomes in Clinical Research Using Multivariate Multilevel Models. J Consult Clin Psychol. 2014;82(5):920–30.
